# Extreme pubic hair removal as a potential risk factor for recurrent urinary tract infections in women

**DOI:** 10.1038/s41598-023-46481-6

**Published:** 2023-11-03

**Authors:** Andrzej Galbarczyk, Urszula M. Marcinkowska, Magdalena Klimek, Grazyna Jasienska

**Affiliations:** 1 Department of Human Behavior, Ecology and Culture, Max Planck Institute for Evolutionary Anthropology, Leipzig, Germany; 2 Department of Environmental Health, Faculty of Health Sciences, Jagiellonian University Medical College, Kraków, Poland

**Keywords:** Urogenital diseases, Urogenital diseases, Risk factors, Infection, Anthropology

## Abstract

Urinary tract infections (UTIs) are the most common infections experienced by women. Previously, scalp and facial hair in men have been shown to inhibit the growth of pathogenic bacteria. Here we hypothesize that having hairy genitalia might protect women from UTI. This study investigated grooming habits and occurrence of UTIs in the past 12 months in 2409 women (aged 18–45). Women who reported removing all their pubic hair at least weekly were defined as extreme groomers (66.8%). We collected additional information on covariates including age, having a first UTI at or before age 15, spermicide use, having a new sex partner, and frequency of sexual intercourse during the past year. Extreme grooming was not associated with the risk of being diagnosed with UTI (OR = 1.17, 95% CI = 0.90–1.52), but was associated with a higher risk of recurrent UTIs, defined as three or more UTIs within 12 months (OR = 3.09, 95% CI = 1.35–7.06), after controlling for age, history of UTIs, and sexual practices. Other studies have found that hygienic purposes are the most common motivations for pubic hair removal. These results suggest that along with their pubes, women may be getting rid of important microbial niche and protection against recurrent UTIs.

## Introduction

Urinary tract infections (UTIs) are one of the most common infections worldwide, especially among women^1^. On average, one out of two women develop at least one UTI during her lifetime^2,3^. Many of them suffer from a so-called recurrent UTIs (rUTIs), when the infection occurs at least three times throughout 1 year^4^. Although most UTIs are caused by one species of bacteria, namely *Escherichia coli*, some of them can be caused by other bacteria or fungi^5^. UTIs cause significant patient distress and significant economic costs to the health care system and become increasingly difficult to treat, owing to the widespread emergence of an array of antibiotic resistance mechanisms^6^. In this study we explore the potential associations between pubic hair removal and the risk of UTI and rUTIs.

Varying forms of pubic hair removal have been practiced across many cultures for centuries^7^. But since the mid-1990s, substantial or total removal of pubic hair has become a common practice in many Western societies^8^. Pubic hair removal is carried out by both men and women but appears to be more common among women^7^. Hygienic purposes are the most common motivations for pubic hair removal^9^. In his 1976 letter to the editor of *Urology*, Han M. Hanafy wrote that he had “noticed there are ‘occasional’ urinary tract infections in the Middle Eastern women compared to ‘frequent’ recurring infections in women in the United States. A striking difference is the customary ‘shaving’ or ‘epilation’ of the pubic-genital hair of married women in the Middle East. Many urologists from different countries in the Middle East concur that there is less infection in women in these countries because they shave or epilate the pubic-genital hair”^10^. However, Hanafy did not support these observations with any data. Actually, we were not able to find any studies demonstrating that pubic hair removal could affect the risk of UTIs.

In general, the idea of hair being dirty and unhygienic seems to be incorrect. Human hair has actually been shown to inhibit the growth of pathogenic bacteria. A study by Wakeam et al.^11^ demonstrated that bearded healthcare workers do not harbor more virulent bacteria in their beards and, in fact, clean-shaven men were 10 percent more likely to harbor colonies of *Staphylococcus aureus* and more than three times as likely to be colonized with methicillin-resistant coagulase-negative staphylococci. Similarly, El Edelbi et al.^12^ has shown that non-bearded healthcare workers had a significantly higher bacterial load in their facial flora than their bearded counterparts. Moreover, men with beards have bacteria that has antibacterial activity that could inhibit the growth of *Escherichia coli* and *Staphylococcus aureus*^13^. Another study demonstrated significant antibacterial effects of human scalp hair shafts on *Staphylococcus aureus* and *Staphylococcus epidermidis*^14^.

Therefore, if the facial and scalp hair has some antimicrobial properties, the same could apply to pubic hair. To the best of our knowledge this is the first study exploring the relationship of pubic hair removal with the risk of UTI and rUTIs among women of reproductive age. Here we hypothesize that having hairy genitalia might protect women from UTI and rUTIs.

## Methods

Our study included adult women aged 18–45 years from Poland. The upper age limit was set at 45 years to avoid the effect of menopause-related hormonal changes that can increase susceptibility to UTIs^15^. Participants completed a cross-sectional online survey (Qualtrics, Provo, UT, USA) advertised via social media (e.g., Facebook). The survey was promoted as a “Women's intimate health and wellness study”. The survey began with a short introduction where the general purpose of the study was explained to the participants and informed consent was obtained. All data were anonymously collected. The study was performed in accordance with the Declaration of Helsinki. Ethics approval was not required for this type of study, in accordance with Polish national legislation^16^.

The questionnaire collected information about age, medical history (age at first UTI, being pregnant and having genitourinary surgery during the past 12 months, being diagnosed with diabetes), and sexual practices (spermicide use, having a new sex partner, and frequency of sexual intercourse during the past 12 months). Participants were also asked to report their pubic hair removal and grooming practices (frequency of grooming and the amount of pubic hair participants typically remove) in the past 12 months. Following the methodology of Luster et al.^17^ we defined extreme groomers as those who remove all their pubic hair via grooming weekly or daily, all other women were classified as non-extreme groomers. Participants were also asked about the occurrence of UTI. UTI was assessed using the question, “During the past 12 months, how many times has a doctor told you that you had a urinary tract infection?”. Women were considered to meet the case definition for rUTIs if they had experienced at least three UTIs episodes in the past 12 months^18^.

Descriptive statistics were used to assess participant characteristics. Two binary logistic regression models were conducted to estimate the risk of being diagnosed with UTI and rUTIs in the past year among extreme groomers and non-extreme groomers. All analyses were a priori controlled for risk factors of UTI: age, having a first UTI at or before 15 years of age (*Yes/No*), spermicide use (*Yes/No*), having a new sex partner (*Yes/No*), and frequency of sexual intercourse (*At least once per month/Less often*) during the past year^15,19^. All analyses were conducted using Statistica version 13.3 (TIBCO Software Inc., Palo Alto, CA, USA). We decided to exclude women who reported being pregnant (n = 144) or having genitourinary surgery (n = 65) during the past 12 months, as well as those diagnosed with diabetes (n = 33), since all those factors could independently influence UTI occurrence^19^.

## Results

The final sample consisted of 2409 women aged 18–45 (mean = 22.7, SD = 5.43). The characteristics of the study participants are presented in Table 1. Most of the participants (n = 2013; 83.6%) reported removing pubic hair via grooming at least weekly. Seventy-four percent of women (n = 1784) declared that they usually removed all their pubic hair in the past 12 months. Nearly 67% of the women (n = 1608) met both criteria and were classified as extreme groomers. Prevalence of at least one diagnosed UTI within the past 12 months was 15.7%, and 2.3% of participants reported rUTIs.

**Table 1:** The characteristics of study participants (N = 2409).

	N	%
At least one UTI in the past year	378	15.69
Recurrent UTIs	56	2.32
Extreme grooming	1608	66.75
New sex partner in the past year	907	37.65
Frequent sexual intercourse (≥ 1 time per month)	1635	67.87
Age at first UTI ≤ 15 years	293	12.16
Spermicide use	207	8.59

Extreme grooming was not associated with the risk of being diagnosed with at least one UTI during the last year (OR = 1.17, 95% CI = 0.90–1.52), after controlling for age, history of UTIs, and sexual practices. In this model frequent sexual intercourse (≥ 1 time per month) and having a first UTI at or before 15 years of age increased the likelihood of being diagnosed with UTI during the last year. Age, spermicide use, and having a new sex partner during the past year were not associated with UTI (Table 2).

**Table 2:** Extreme grooming and the likelihood of urinary tract infections (UTIs) and recurrent urinary tract infections (rUTIs), after controlling for participant’s age, history of UTIs, and sexual practices. Results in bold are statistically significant (p < 0.05).

	Urinary tract infections				Recurrent urinary tract infections			
	OR	− 95%	+ 95%	p	OR	− 95%	+ 95%	p
Age (years)	1.00	0.98	1.02	0.797	0.98	0.93	1.04	0.559
Extreme grooming	1.17	0.90	1.52	0.239	**3.09**	**1.35**	**7.06**	**0.007**
New sex partner in the past year	0.86	0.68	1.10	0.231	**0.54**	**0.30**	**0.97**	**0.038**
Frequent sexual intercourse (≥ 1 time per month)	**3.05**	**2.24**	**4.16**	**< 0.001**	**8.73**	**2.66**	**28.60**	**< 0.001**
Age at first UTI ≤ 15 years	**3.63**	**2.74**	**4.80**	**< 0.001**	**3.05**	**1.65**	**5.64**	**< 0.001**
Spermicide use	0.98	0.66	1.43	0.899	1.12	0.49	2.54	0.785

However, women who were extreme groomers within the past 12 months were three times more likely to have had recurrent UTIs (OR = 3.09, 95% CI = 1.35–7.06), after controlling for age, history of UTIs, and sexual practices (Table 2). Having a first UTI in childhood, having a new sex partner, and frequent sexual intercourse during the past year were associated with the likelihood of recurrent UTIs. Age and spermicide use were not associated with recurrent UTIs (Table 2). The unadjusted odds ratios are presented in Supplementary Table S1.

## Discussion

Our findings suggest that extreme pubic hair grooming may be associated with rUTIs among women of reproductive age. There are several evolutionary hypotheses on why pubic hair exists, but none of them is supported by evidence^9,20^. Here we propose a new explanation—pubic hair might be beneficial for maintaining women’s urogenital health. Presented results suggest that along with their pubes, women may be getting rid of important protection against rUTIs. There are several plausible mechanisms which may explain our findings.

It could be hypothesized that the microbial communities that reside in pubic hair play key roles in protecting women’s urogenital health. Pubic hair is somewhat insulated from the environment and is colonized with a niche-specific bacteria^21^. Therefore, pubic hair microbiome seems to be quite stable (to the extent that bacterial profiling can be used in forensic practice). In contrast to male pubic hair, female pubic hair harbors different *Lactobacillus* species that could confer ‘antimicrobial protection’ by preventing colonization by other microorganisms^21^. Microbial populations of *Lactobacillus* species have a strong inhibitory effect on *Escherichia coli*, the most common causative agent for UTI^22^.

Apart from being a microbial niche for beneficial bacteria, pubic hair might exhibit antimicrobial properties of their own: hair-derived proteins and peptides with antimicrobial activity towards bacteria and fungi have been previously identified in human hair shafts^23,24^. It therefore seems plausible that extreme pubic hair removal may negatively affect the urogenital microbiome colonization.

However, only one recent study examined whether the urogenital microbiome differs between women with and without pubic hair. In this relatively small but cleverly designed study, urogenital microbiome was analyzed in women with and without pubic hair and in the same woman when she changed her pubic hair status^25^. This study has shown that pubic hair status did not determine women’s baseline genitourinary microbiome, but changing their pubic hair status—removing all or growing out pubic hair—influenced the composition of the vaginal microbiome (however not the urinary microbiome). Another study has shown that microbial sharing between the vaginal and bladder microbiota is very common and not only limited to pathogens but is also characteristic of health-associated commensals^26^. Geynisman-Tan et al.^25^ attributed the lack of changes in the urinary microbiome after changing pubic hair status to the fact that it could take more than 1 month to change the vaginal microbiome and to see a reciprocal change in the urinary microbiome. Further research is certainly needed to confirm these findings.

Finally, it should be noted, that pubic hair removal can lead to many health complications. The most common is genital itching, followed by irritation, skin infection, rash, cut or bleeding, acne, allergic reaction, and ingrown hair^27^. It has been suggested that pubic hair removal may cause skin microtrauma and subsequent spread of infectious agents throughout the skin in the genital area^28^. A vast majority of complications related to pubic hair removal occurs among women who shave pubic hair with a razor^29^. Unfortunately, we have not collected the information about the methods used to remove pubic hair. Certainly, more detailed information should be collected in future studies. Extreme and high-frequency pubic hair removal was also positively associated with a history of self-reported Sexually Transmitted Infections (STIs), including herpes, human papillomavirus, syphilis, and molluscum^30^ (but see^17,31^).

This study has some limitations to consider. Firstly, this was a fairly homogenous sample of young Polish women, the majority of whom were white. This was a convenience sample, and the results may not be generalizable. Future research is needed to assess the pubic hair removal practices and UTIs prevalence in more diverse populations. Secondly, the data in our study were self-reported, prone to memory bias and could not be clinically verified. Nevertheless, basing our research on a self-report internet survey resulted in a large sample size, which allowed to track the rUTIs prevalence (which is far less common than the typical UTI). Finally, future studies should test whether the relationship we observed can depend on the other perineal hygiene habits^32^ and medication, probiotics or natural products use^33^. Unfortunately, we did not collect such data.

To summarize, we propose a simple solution to a very important public health problem. It appears that giving up the extreme pubic hair removal will not completely prevent women from the occurrence of urinary tract infections. But despite the fact, that extreme pubic hair removal is commonly considered by many to be required to meet society’s standards of attractiveness, femininity, and cleanliness among women^28,34^ our results suggest that especially women experiencing recurrent UTIs might let their pubes grow and embrace hairy genitalia.

### Supplementary Information

Supplementary Table S1.

## Data Availability

The questionnaire and the datasets used and analyzed during the current study are available from the corresponding author on reasonable request.
