# Supplementary material for: Extreme pubic hair removal as a potential risk factor for recurrent urinary tract infections in women
Source: Sci Rep. 2023 Nov 3;13:19045. doi: 10.1038/s41598-023-46481-6 (PMC10624866; doi:10.1038/s41598-023-46481-6)
Supplement: Supplementary file 1 — Supplementary Table S1. [file 41598_2023_46481_MOESM1_ESM.pdf]

Extreme pubic hair removal as a potential risk factor for recurrent urinary tract infections in women

Andrzej Galbarczyk, Urszula M. Marcinkowska, Magdalena Klimek, Grazyna Jasienska

Table S1. The unadjusted odds ratios for urinary tract infections and recurrent urinary tract infections.

	**Urinary tract infections**				**Recurrent urinary tract infections**			
	OR	-95%	+95%	p	OR	-95%	+95%	p
**Age (years)**	1.02	1.00	1.04	0.071	0.99	0.95	1.05	0.843
**Extreme grooming**	1.26	0.99	1.60	0.063	**3.57**	**1.61**	**7.91**	**0.002**
**New sex partner in the past year**	1.00	0.79	1.25	0.971	0.72	0.40	1.27	0.256
**Frequent sexual intercourse (≥1 time per month)**	**2.87**	**2.15**	**3.84**	**<0.001**	**8.61**	**2.68**	**27.6**	**<0.001**
**Age at first UTI ≤15 years**	**3.36**	**2.56**	**4.41**	**<0.001**	**2.73**	**1.49**	**5.00**	**0.001**
**Spermicide use**	1.23	0.85	1.78	0.271	1.54	0.69	3.44	0.295
